# Trends in the prevalence and incidence of orphanhood in children and adolescents <20 years in rural KwaZulu-Natal South Africa, 2000-2014

**DOI:** 10.1371/journal.pone.0238563

**Published:** 2020-11-24

**Authors:** Gabriela Mejia-Pailles, Ann Berrington, Nuala McGrath, Victoria Hosegood

**Affiliations:** 1 Independent Consultant, Mexico City, Mexico; 2 Department of Social Statistics & Demography, University of Southampton, Southampton, United Kingdom; 3 Department of Population Sciences & Primary Care, University of Southampton, Southampton, United Kingdom; 4 Africa Health Research Institute, KwaZulu-Natal, South Africa; Johns Hopkins Bloomberg School of Public Health, UNITED STATES

## Abstract

**Background:**

In South Africa, large increases in early adult mortality during the 1990s and early 2000s have reversed since public HIV treatment rollout in 2004. In a rural population in KwaZulu-Natal, we investigate trends in parental mortality and orphanhood from 2000–2014.

**Methods:**

Using longitudinal demographic surveillance data for a population of approximately 90,000, we calculated annual incidence and prevalence of maternal, paternal and double orphanhood in children and adolescents (<20 years) and, overall and cause-specific mortality of parents by age.

**Results:**

The proportion of children and adolescents (<20 years) for whom one or both parents had died rose from 26% in 2000 to peak at 36% in 2010, followed by a decline to 32% in 2014. The burden of orphanhood remains high especially in the oldest age group: in 2014, 53% of adolescents 15–19 years had experienced the death of one or both parents. In all age groups and years, paternal orphan prevalence was three-five times higher than maternal orphan prevalence. Maternal and paternal orphan incidence peaked in 2005 at 17 and 27 per 1,000 person years respectively (<20 years) before declining by half through 2014. The leading cause of parental death throughout the period, HIV/AIDS and TB cause-specific mortality rates declined substantially in mothers and fathers from 2007 and 2009 respectively.

**Conclusions:**

The survival of parents with children and adolescents <20 years has improved in tandem with earlier initiation and higher coverage of HIV treatment. However, comparatively high levels of parental deaths persist in this rural population in KwaZulu-Natal, particularly among fathers. Community-level surveillance to estimate levels of orphanhood remains important for monitoring and evaluation of targeted state welfare support for orphans and their guardians.

## Introduction

In all societies, the death of a parent during childhood and adolescence, especially the death of a mother, is widely acknowledged as having potentially profound consequences on child health and wellbeing [1–8]. Studies in South Africa conducted since the start of the HIV epidemic have shown orphaned children to be at increased risk of poorer health and educational outcomes compared to other children (See for example, [9–11]). The severity and duration of negative consequences are influenced by the type, cause and timing of orphaning. For example, there is consistent evidence that double or maternal orphans are at greater risk than paternal orphans [2,12,13]. Compared to other causes of parental death, children experiencing the death of a parent due to AIDS or injuries have higher risks of poorer child mental health [14] and, significant changes in their living arrangements due to household dissolution and migration [14–16].

In rural KwaZulu-Natal, South Africa, men and women become parents in a context of very high HIV population prevalence [17,18]. In the early 2000s, forecasts based on population data suggested that even if HIV incidence remained stable, in the absence of public HIV treatment, between 24% and 33% of children would go on to become maternal and paternal orphans by their 18^th^ birthday [19,20]. When antiretroviral treatment (ART) was rolled out from 2004 onwards, overall adult mortality rates declined reflecting reduced adult mortality due to HIV/AIDS [21–23]. Consequently, the previously forecasted level of orphan incidence are no longer expected even in communities that experienced severe HIV epidemics and where the prevalence of HIV is still increasing due to the improved survival of people living with HIV. However, there are very few recent and detailed published population orphanhood estimates for South Africa, especially of orphan incidence rates. Given that orphans and vulnerable children (OVC) are a key population group targeted by statutory and private support services [24–27]; uncertainty about recent changes in the numbers and types of orphans in HIV affected communities is a challenge for planning relevant and effective health and welfare-related policy responses.

This paper contributes to filling this gap with robust estimates of the levels and trends in age-specific orphan prevalence and incidence in rural KwaZulu-Natal over the period 2000–2014. We are able to situate these new estimates of orphanhood in the context of changes in the level and pattern of adult mortality documented by previously published studies from the same area [9,15,19,21,28–31]. We describe changes over the 15 year period (2000–2014) in the ages at which children are orphaned, different types of orphans, children’s exposure to different causes of parental mortality. We discuss the implications of these recent trends in orphaning for programme and research activities focusing on orphans and other children affected by HIV in South Africa.

## Methods and data

### Study site and setting

We analysed longitudinal, population-based demographic and health surveillance data available in the Africa Centre Demographic Information System (ACDIS) for the period 2000–2014. The population data were collected prospectively in approximately 11,000 households resident in part of the Umkanyakude district in northern KwaZulu-Natal, South Africa. The Zulu-speaking communities in this predominantly rural area are broadly similar to many other rural parts of the province where governance is a mixture of tribal and municipal authorities. Waged employment and government grants are the main source of household income rather than agriculture. The level of local unemployment in the study area is high and consequently many men and women migrate to other places within South Africa in order to work or seek work [28,32]. Substantive proportions of adults and children belonging to households in rural KwaZulu-Natal are circular migrants [33]. Circular migration is a long-established form of oscillatory mobility involving households in multiple rural or urban locations and work sites. In South Africa, the apartheid migrant labour system deeply entrenched the importance of circular migration for household livelihoods and family functioning [34]. Typically circular migrants remain socially and physically connected through visits, involvement and shared financial and material support [35]. It is also common for children living in rural areas to experience periods of parental absence and caregiving by non-biological parents, due both to parental migration and low rates of marriage and cohabitation of parents [36,37].

The severe HIV epidemic experienced in KwaZulu-Natal since the end of the 1990s has been well-described elsewhere (See for example, [21,38]). Calculated using data from routine, HIV testing surveys conducted within the surveillance system study population, estimates of HIV prevalence between 2003 and 2014 in resident men and women (15–49 years) increased from 21% (20.9–22.7) in 2003/4, to 33% in 2014 [38,39]. In the study area, public access to HIV treatment has been delivered through the Hlabisa HIV Treatment and Care Programme of the South African Department of Health (Hlabisa HTCP) [40]. Between 2000 and 2014, public ART in the study area can be broadly characterised into two periods: 2000–2005 in which public ART was unavailable or beginning to be rolled out across primary health care (PHC) clinics in the local health district; and, 2006 onwards when public ART was widely available and where treatment could be initiated at several local PHC clinics [40,41]. In the same heath district, estimated population ART coverage in HIV-positive adults was 31.7% in 2012 and 45% in 2017 [38,42].

### Study population data

The study population comprises approximately 90,000 members of households in the study area. ACDIS procedures and data collection systems are described in detail elsewhere [33,43,44]. In brief, from January 2000 data on all resident and non-resident household members were collected every 3–4 months, including all new births, deaths and migrations. When an adult or child death is recorded, a verbal autopsy (VA) interview is conducted within 3 to 10 months with the deceased family or carers. During the interview additional information about the death is recorded in order that a cause of death can be assigned using InterVA-4 software [29]. In 2016, the ACDIS study area and data collection activities were incorporated into a new Population Intervention Platform Study Area (PIPSA) conducted by the Africa Centre Health Institute (AHRI).

### Parental data

During routine household visits, household respondents are asked to confirm or update the survival status of biological mothers and fathers for every resident and non-resident household member. In cases where the biological parent has also been registered in ACDIS, typically when the parent is or has been a member of the same household as his or her child, the parent’s and child’s data are linked in the surveillance system. Linked biological parent-child records provide researchers with another, indirect source of longitudinal data from which to ascertain if, and when the parent died. An exception was the period between the end of 2000 and January 2004 during which questions about the survival status of unlinked, biological parents were not asked at every household visit. However, using other information we were able to reconstruct survival histories of most parents with gaps in prospective data collection.

In this study, we developed a set of pre-analysis validation criteria to guide the detection and adjustment of inconsistencies across repeated observations of the survival status of biological mothers and fathers. Increasing with age due to longer periods of follow up, most children and adolescents have repeated observations of parental survival status. For example, an average of 20 maternal orphan status observations are available for resident children aged 17 years. Of children with multiple reports of parental survival status, less than 3% had inconsistencies between two or more observations. We examined inconsistent observations to reconcile information where possible using detailed individual data on parents or subsequent reports by household respondents. In the estimates, unlinked parents for whom no survival information was available were included with dead parents. This approach is similar to that used by international agencies and some researchers to calculate estimates of orphan prevalence from cross-sectional survey data [45]. In non-conflict settings in Africa, it unusual for household respondents not to know or want to say whether a child’s mother or father is alive or dead. Thus, it has been argued that when information on a parent is not known, the parent is effectively unavailable to his or her child [24]. After pre-analyses validation for inconsistencies in data, less than one percent of all children and adolescents had no valid observation of maternal or paternal status. These children were not included our analyses (see S1 Table footnote for details of the number of missing children and adolescents in each year).

### Measures

#### Categorising orphans

The term ‘orphan’ is variously conceptualised in the global literature with a wide range of different definitions used in descriptions and measurement of orphanhood [27]. In this study, we define four non-overlapping types of orphan: *Non-orphan—*both biological parents are alive. *Maternal orphan—*mother is dead or her survival is not known, father is alive. *Paternal orphan—*father is dead or his survival is not known, mother is alive. *Double orphan*: mother and father are dead or survival status unknown. We also include a summary measure of whether ‘one or both parents are dead’ as this measure is often included in survey and national reports. All measures of orphaning presented in this paper are for both sexes combined as we found no significant differences in the levels or patterns of orphanhood in girls and boys. In this paper, we use an upper age limit of 19 years. Older South African youth have a high degree of dependency of on financial and material support from family given that the majority attend school until 18 or 19 years and, over half of all school leavers in this community are unemployed [9,32,46]. Furthermore, commentators have highlighted knowledge gaps caused by the omission of older adolescents in orphanhood statistics and programmes in sub-Saharan Africa [47].

#### Prevalence and incidence of orphanhood

Annual orphan prevalence estimates were calculated for all children and adolescents (<20 years) resident in the study area on the 31^st^ July (mid-year population). Prevalence estimates were calculated for the four types of orphan separately (maternal, paternal, double and non-orphan) and for a combined measure (one or more parents are dead). Maternal and paternal orphaning incidence were estimated as the number of maternal or paternal deaths per 1,000 person (i.e. child) years observed (PYO) among resident children (<20 years) whose mothers and father were alive at the start of the follow-up period. Observed years were censored for children that died, migrated outside the demographic surveillance area or ended their household membership before the end of 2014. For children whose parents died during the observation period, exposure was estimated as the time between the start of follow-up and the date of death of the parent. The time of exposure for these children was censored at the time when they were last observed. We present the orphan incidence data for four years within the follow-up period: 2000, 2005, 2010 and 2014.

#### Causes of parental death

Parental deaths were assigned to one of five groups of causes: HIV/AIDS and TB, communicable, non-communicable, injury and undetermined [29]. In this paper, we consider deaths due to HIV/AIDS and TB together to reflect the high levels of co-morbidity of HIV and TB in the study community, as well as the similarity of associated symptoms. For mothers and fathers of children in the study population, we estimated the annual all-cause mortality rates for the years 2000–2014 and the relative contribution of the five groups of causes of death to overall mortality. When calculating cause-specific mortality each deceased parent was included only once. Given the different age distribution of deceased mothers and fathers and, the marked differences in cause-specific mortality at older ages, we disaggregated the cause-specific mortality data further and present the results separately for mothers in two age groups: <35 years and 35+ years, and fathers aged: <40 years and 40+ years.

In order to examine whether the rate at which children and adolescents experience different cause-specific maternal or paternal deaths changes with age or over the period before and after ART, we calculated the maternal and paternal incidence for the years 2000, 2005, 2010 and 2014, by age group and cause of death category. We also calculated annual cause-specific incidence estimates for maternal and paternal orphaning in years 2000, 2005, 2010 & 2014. Stata software was used for all statistical analyses (Version 13, StataCorp).

### Ethics approval

Ethics approval for the demographic surveillance study and analyses of these data was given by the College of Health Sciences Biomedical Research Ethics Committee, University of KwaZulu-Natal, South Africa. The study project received approval from the research ethics committee of the University of Southampton: ID:9841.

## Results

**Fig 1:**
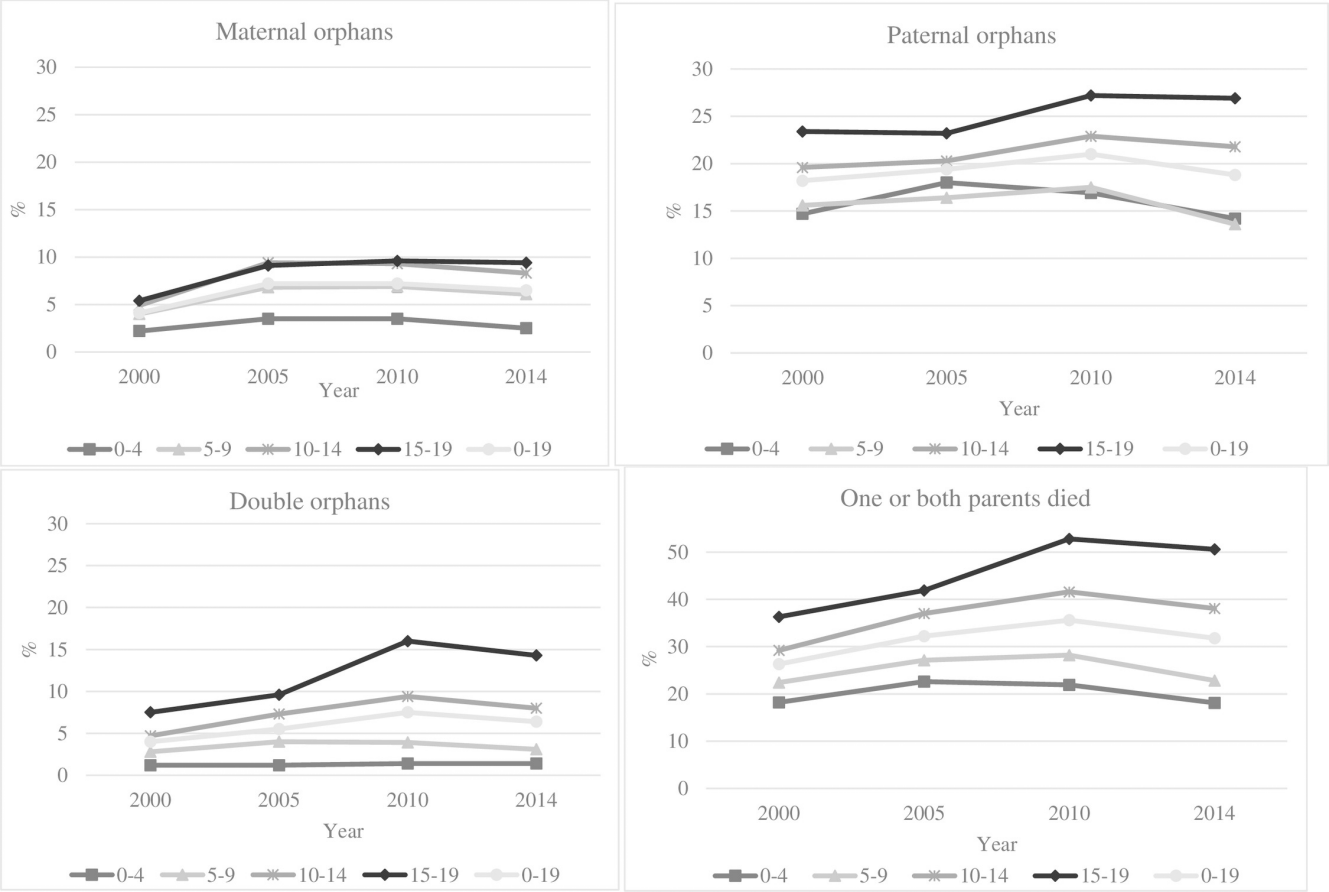
Orphanhood prevalence by orphan status and age group in children and adolescents <20 years in 2000, 2005, 2010, 2014.

The levels and trends in age-specific maternal, paternal and double orphan prevalence are presented in Fig 1 for the years 2000, 2005, 2010 and 2014 (S1 Table presents additional detailed data related to Fig 1 including absolute numbers, estimates and 95% confidence intervals). The proportion of children and adolescents (<20 years) for whom one or both parents had died rose from 26% in 2000 to peak at 36% in 2010, followed by a decline to 32% in 2014. In all age groups and years, the level of paternal orphan prevalence was three-five times higher than maternal orphan prevalence. The pattern of maternal orphan prevalence rose from 2000 to plateau between 2005 and 2010, declining slightly by 2014. In contrast, paternal orphan prevalence continued to rise through 2010—declining later and more slowly than maternal orphan prevalence. The largest difference between paternal and maternal orphan prevalence was in the youngest age group (0–4 years). In this age group, the seven-fold difference in 2000, 15% and 2% respectively, remained very similar across the period. Orphaning is a cumulative experience with age, with the oldest age group of children (15–19 years) experiencing the highest prevalence of every orphan type. By 2010, 53% of children aged 15–19 years had experienced the death of one or both parents; with 10% in this age group being maternal orphans and 27% paternal orphans. Increases in double orphaning among older age groups of children were very pronounced through 2010; more than doubling from 7.5% (2000) to 16% (2010) in 15–19 year olds. By the end of the study period in 2014, the prevalence of all orphan types had declined close to, but had not yet reached, the lower levels observed at the start of data collection in 2000.

**Fig 2:**
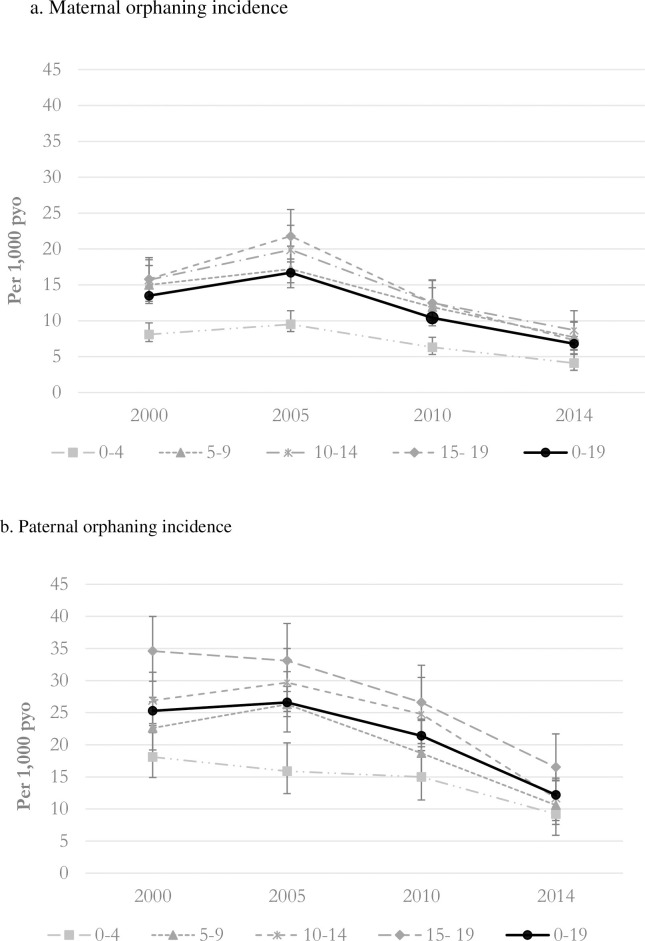
Maternal and paternal orphaning incidence (per 1,000 PYO) by orphan status and age group in children and adolescents <20 years, 2000, 2005, 2010, 2014.

Fig 2 presents age-specific incidence of maternal and paternal orphaning (S2 Table presents additional detailed data related to Fig 2 including numbers and 95% confidence intervals). Estimates of maternal and paternal orphaning incidence (age <20 years) peaked in 2005, 17 and 27 per 1,000 person years respectively. As with orphan prevalence, the difference between maternal orphaning incidence and the higher rates of paternal orphaning incidence is greatest in the youngest children (0–4 years). Across the period, the two oldest age groups (10–14 and 15–19 years) experience the highest incidence of maternal and paternal orphaning. The highest level of paternal orphaning incidence estimated in these age groups was in 2005, 30 and 33 per 1,000 person years respectively. Compared with orphan prevalence, orphaning incidence declined earlier and more rapidly; by 2014, the incidence of all types of orphan was less than half those in 2000.

### Causes of parental death

**Fig 3:**
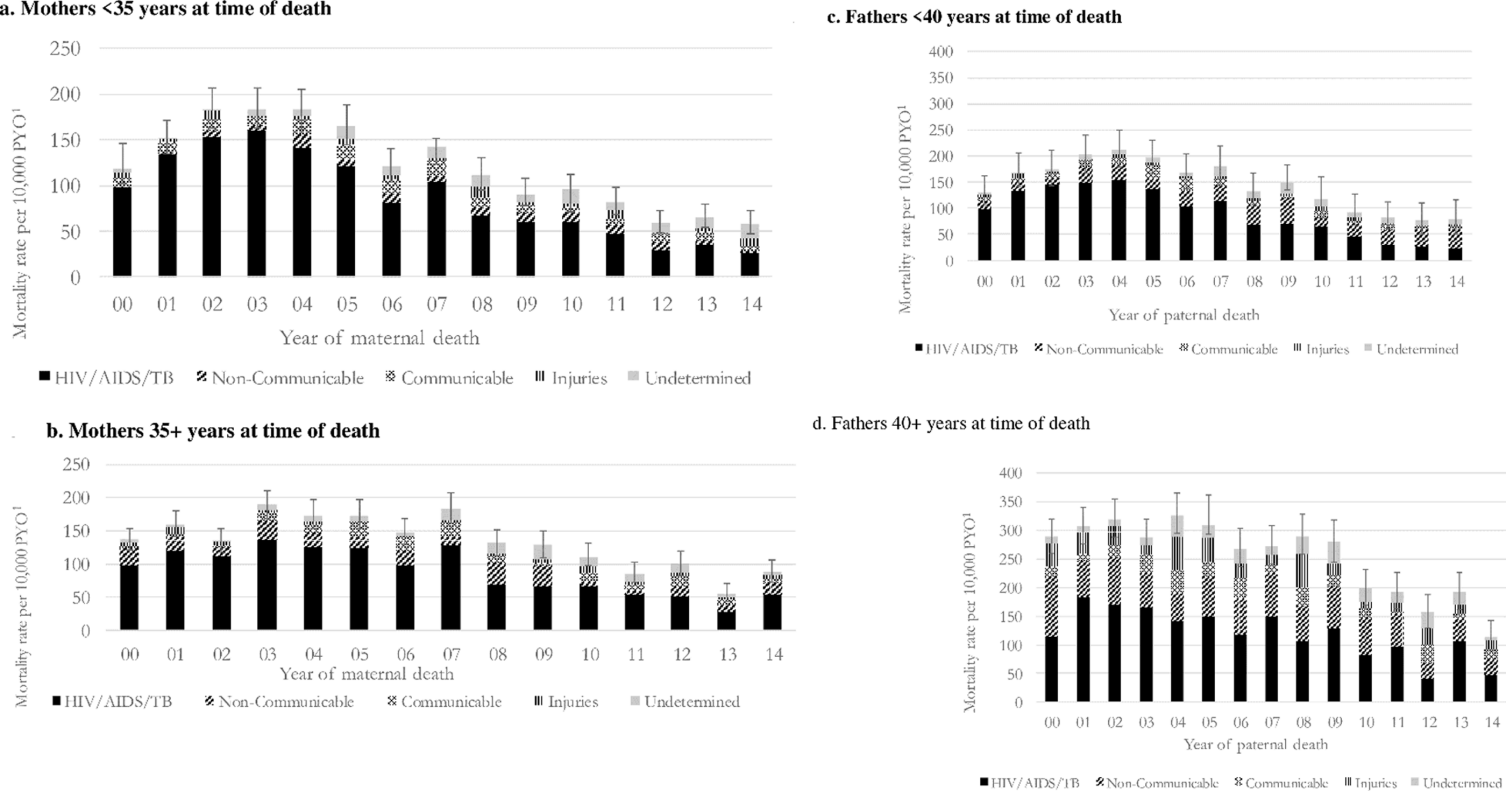
Mortality rates per 10,000 PYO by year (2000–2014), overall and by cause of death, among deceased mothers (Fig 3A aged <35 years and Fig 3B aged 35+ years) and deceased fathers (Fig 3C aged <40 years and Fig 3D aged 40+ years) of children and adolescents <20 years. a. Person years observed (PYO) 2000–2014 = 306,729; Number of maternal deaths <35 years 2000–2014 = 3,896. b. Person years observed (PYO) 2000–2014 = 132,477; Number of maternal deaths 35+ years 2000–2014 = 1,710. c. Person years observed (PYO) 2000–2014 = 89,928; Number of paternal deaths <40 years 2000–2014 = 1,547. d. Person years observed (PYO) 2000–2014 = 139,041; Number of paternal deaths 40+ years 2000–2014 = 3,659.

Fig 3A–3D show the trends in annual cause-specific mortality in mothers and fathers by age. HIV/AIDS and TB was the dominant cause of parental mortality throughout the period. Substantial reductions in the rates of mothers and fathers dying due to HIV/AIDS and TB occurred after 2007 and 2009 respectively. The cause-specific mortality of older mothers (35+ years at death) closely resembles younger mothers (<35 years at death) with HIV/AIDS and TB remaining the largest category of mortality across the period 2000–2014. In contrast, at the beginning of the period and most years from 2008, a larger proportion of deaths in older fathers (40+ years at death) were attributable to non-communicable diseases and injuries than HIV/AIDS and TB. For younger fathers (<40 years at death), the rate of deaths due to HIV/AIDS and TB only fell below those due to non-communicable diseases and injuries several years later, from 2012.

Table 1 shows the estimated incidence of maternal and paternal orphaning overall and by separate age groups for each cause of death in 2000, 2005, 2010 and 2014. The incidence of maternal and paternal orphaning due to HIV/AIDS and TB reduced by more than half over the period in all age groups of children. The exception was the slightly smaller decline in the incidence rate of paternal orphaning due to HIV/AIDS and TB in the oldest age group (15–19 years). In 2000, at the beginning of the period the age-specific rate of orphaning was much higher in this age group, 14.5 per 1,000 person years, than in younger age groups. Rates of paternal orphan incidence due to injury-related deaths were more than four-fold higher than maternal orphan incidence. The overall incidence of each cause of parental death declined across the period. In contrast, the incidence of orphaning due to communicable diseases and undetermined causes increased slightly; more so with respect to deaths of mothers than fathers.

**Table 1:** Cause-specific maternal and paternal orphaning incidence rates per 1000 PYO in children and adolescents <20 years by age group in 2000, 2005, 2010, 2014.

Cause of death grouped	Maternal orphaning incidence^1^^,^^2^				Paternal orphaning incidence			
**Age group (years)**	**2000**	**2005**	**2010**	**2014**	**2000**	**2005**	**2010**	**2014**
**HIV/AIDS and TB**								
**0–4**	6.6 (5.2–8.4)	6.6 (5.1–8.6)	3.9 (2.8–5.4)	1.7 (1.0–2.9)	8.6 (6.5–11.4)	8.9 (6.4–12.4)	5.8 (3.7–8.9)	2.9 (1.3–6.5)
**5–9**	11.8 (9.8–14.2)	12.7 (10.5–15.4)	7.2 (5.5–9.3)	3.7 (2.6–5.3)	12.8 (10.2–15.9)	12.9 (10.1–16.7)	8.3 (5.8–12.0)	4.0 (2.4–7.0)
**10–14**	11.9 (9.8–14.3)	13.8 (11.4–16.7)	7.1 (5.3–9.5)	4.6 (3.2–6.7)	12.4 (9.9–15.6)	12.6 (9.8–16.2)	11.6 (8.6–15.7)	4.7 (2.8–8.2)
**15–19**	9.5 (7.6–11.8)	15.2 (12.6–18.4)	8.2 (6.2–10.8)	3.7 (2.4–5.7)	14.5 (11.6–18.2)	15.5 (12.3–19.5)	10.5 (7.7–14.4)	9.0 (6.2–13.1)
**0–19**	9.9 (8.9–11.0)	11.8 (10.7–13.1)	6.3 (5.5–7.3)	3.3 (2.7–4.1)	12.1 (10.7–13.5)	12.6 (11.1–14.3)	9.1 (7.7–10.8)	5.4 (4.2–7.0)
**Injuries**								
**0–4**	0.3 (0.1–0.9)	0.3 (0.1–1.0)	0.3 (0.1–1.0)	0.4 (0.1–1.1)	5.2 (3.6–7.4)	1.5 (0.7–3.3)	4.0 (2.4–6.8)	2.4 (1.0–5.8)
**5–9**	0.6 (0.3–1.4)	0.3 (0.1–1.0)	0.5 (0.2–1.4)	1.0 (0.5–2.0)	3.2 (2.1–5.0)	3.7 (2.3–5.9)	3.5 (2.0–6.1)	4.0 (2.4–7.0)
**10–14**	1.1 (0.6–2.0)	0.3 (0.1–1.1)	1.2 (0.6–2.5)	0.7 (0.3–1.8)	5.1 (3.6–7.3)	4.8 (3.2–7.2)	2.5 (1.3–4.8)	2.9 (1.5–5.8)
**15–19**	0.6 (0.3–1.5)	0.1 (0.0–1.0)	1.4 (0.8–2.8)	0.4 (0.1–1.4)	4.3 (2.9–6.5)	4.8 (3.2–7.3)	1.9 (0.9–4.0)	1.9 (0.9–4.3)
**0–19**	0.7 (0.4–1.0)	0.3 (0.1–0.5)	0.8 (0.5–1.2)	0.6 (0.4–1.0)	4.5 (3.7–5.4)	3.8 (3.0–4.8)	2.9 (2.2–4.0)	2.9 (2.0–4.1)
**Non-communicable**								
**0–4**	0.6 (0.3–1.4)	0.3 (0.1–1.0)	1.0 (0.5–1.8)	0.1 (0.0–0.9)	3.3 (2.1–5.1)	2.2 (1.2–4.3)	2.0 (1.0–4.2)	1.9 (0.7–5.2)
**5–9**	1.2 (0.6–2.1)	1.0 (0.5–2.0)	0.8 (0.4–1.7)	0.6 (0.3–1.5)	5.3 (3.8–7.5)	3.7 (2.3–5.9)	3.7 (2.2–6.4)	0.3 (0.0–2.2)
**10–14**	1.6 (1.0–2.7)	2.4 (1.5–3.8)	0.9 (0.4–2.1)	1.9 (1.0–3.4)	7.5 (5.6–10.0)	6.3 (4.4–9.0)	7.2 (4.9–10.5)	1.8 (0.8–4.4)
**15–19**	3.6 (2.5–5.1)	2.4 (1.5–3.8)	1.1 (0.5–2.4)	2.0 (1.1–3.5)	13.2 (10.5–16.7)	7.2 (5.1–10.1)	8.6 (6.1–12.2)	3.6 (2.0–6.4)
**0–19**	1.7 (1.3–2.1)	1.5 (1.1–1.9)	0.9 (0.6–1.4)	1.0 (0.7–1.5)	7.2 (6.2–8.3)	4.9 (4.0–6.1)	5.5 (4.4–6.8)	1.9 (1.2–2.9)
**Communicable^3^**								
**0–4**	0.4 (0.2–1.1)	1.2 (0.7–2.2)	0.2 (0.1–0.8)	0.4 (0.1–1.1)	0.7 (0.3–1.8)	1.2 (0.5–3.0)	1.2 (0.4–3.1)	1.5 (0.5–4.5)
**5–9**	0.8 (0.4–1.7)	1.6 (1.0–2.8)	1.3 (0.7–2.4)	1.0 (0.5–2.0)	0.8 (0.3–1.9)	1.5 (0.7–3.2)	1.2 (0.4–3.1)	0.9 (0.3–2.9)
**10–14**	0.8 (0.4–1.6)	2.4 (1.5–3.8)	1.4 (0.7–2.7)	0.9 (0.4–2.0)	1.2 (0.6–2.4)	1.7 (0.8–3.3)	0.8 (0.3–2.6)	1.5 (0.5–3.9)
**15–19**	0.9 (0.4–1.8)	1.7 (1.0–3.0)	1.1 (0.5–2.4)	0.5 (0.2–1.7)	1.5 (0.8–3.0)	1.7 (0.9–3.5)	1.9 (0.9–4.0)	0.6 (0.2–2.6)
**0–19**	0.7 (0.5–1.0)	1.7 (1.3–2.2)	0.9 (0.6–1.4)	0.7 (0.4–1.1)	1.0 (0.7–1.5)	1.6 (1.1–2.2)	1.3 (0.8–2.0)	1.1 (0.6–1.9)
**Undetermined**								
**0–4**	0.1 (0.0–0.7)	0.7 (0.3–1.5)	1.0 (0.5–1.8)	1.5 (0.8–2.6)	0.3 (0.1–1.4)	1.5 (0.7–3.3)	1.7 (0.8–3.9)	0.5 (0.1–3.4)
**5–9**	0.4 (0.2–1.1)	1.6 (1.0–2.8)	2.0 (1.2–3.2)	1.1 (0.6–2.1)	0.5 (0.2–1.5)	2.2 (1.2–4.0)	1.7 (0.8–3.8)	1.2 (0.5–3.3)
**10–14**	0.2 (0.1–0.9)	0.7 (0.3–1.6)	1.9 (1.1–3.3)	0.5 (0.0–0.0)	0.7 (0.2–1.8)	3.1 (1.9–5.2)	2.2 (1.1–4.4)	0.0 (0.0–0.0)
**15–19**	1.4 (0.7–2.4)	1.3 (0.7–2.4)	0.6 (0.2–1.7)	0.7 (0.3–1.9)	0.9 (0.4–2.3)	2.4 (1.3–4.3)	3.8 (2.2–6.4)	1.3 (0.5–3.4)
**0–19**	0.5 (0.3–0.8)	1.0 (0.7–1.5)	1.3 (1.0–1.8)	1.0 (0.7–1.5)	0.6 (0.4–1.0)	2.3 (1.7–3.2)	2.4 (1.7–3.3)	0.8 (0.4–1.6)

^1^ Maternal (or paternal) orphaning incidence is estimated for the year period for resident children <20 years whose mothers (or fathers) were alive at the start of the year; and is expressed 1000 person (i.e.child) years of observation.

^2^ (95% confidence intervals).

^3^ Deaths due attributed to a communicable disease other than HIV/AIDS and TB.

## Discussion

Our analyses of long-term trends over the pre- and post-ART periods (2000–2014) show recent declines in maternal and paternal orphan prevalence and incidence. Having already reached high levels by the start of the period (2000), the proportions of children orphaned continued to rise further, only starting to decline after 2010. Despite huge progress in scaling up the HIV treatment programme since the mid-2000s, these longitudinal population-based data provide evidence that the experience of a parents during childhood or adolescents remained very common in this rural South African community. In 2014, a third (32%) of all children and adolescents (0–19 years) and half (51%) of the older adolescents (15–19 years) had experienced the death of one or both parents respectively. As expected given the broad similarity of social and economic characteristics and public services in the study area to rural communities elsewhere in rural KwaZulu-Natal; these estimates of orphanhood mirror provincial those from national Census and survey data [9,28,48]. However, compared with other South African provinces, KwaZulu-Natal has higher levels of orphanhood. In 2011, the province had more than double the number of single and double orphans (<18 years) then the next highest province—Eastern Cape based on the 2011 Census data [49].

The peak levels of paternal and double orphan prevalence (<20 years) in the study population 2010 (S1 Table) are much higher than the national estimates in the 2011 Census: 21% vs 15% and 7% vs 4% respectively [49]. Whereas estimates of maternal orphan prevalence (<20 years) was the same in the study and nationally (7%). In KwaZulu-Natal, exceptionally high levels of excess male mortality in young adults have been documented at different stages of the HIV epidemic. The causes of excess male mortality particularly in young men, is not only due to HIV/AIDS and TB deaths but also higher rates of road traffic accidents, intentional injuries and non-communicable diseases [20,50,51].

A strength of the directly observed data available from longitudinal population surveillance is that it enables us to estimate and monitor trends in orphan incidence, a much rarer indicator. The levels of both orphan incidence and prevalence that we report for the early period before public ART rollout are very similar to the estimates and forecasts of orphanhood reported on the basis of studies conducted in the early 2000s in the same population [19] and, in KwaZulu-Natal [52,53]. However, after ART rollout, our study shows that there was an immediate decline in orphan incidence but not orphan prevalence. The trend in orphan incidence closely mirrors the timing of reversals in adult mortality reported in the study population [21,23]. In contrast, orphan prevalence is a cumulative indicator and therefore, is less responsive than orphan incidence to immediate impacts of HIV treatment or other factors that improve parental survival. As discussed by Reniers et al (2017), recent declines in adult mortality due to HIV/AIDS is not only a consequence of ART roll-out but of long-term declines in HIV incidence and improvements in coverage and effectiveness of HIV treatment and care programmes [23]. In the study population, estimated HIV incidence in women and men declined year on year from 2014 and 2012 respectively [38].

Understanding the trends in survival of HIV-infected parents in the period post-ART rollout is complicated by changes in treatment access and, testing and treatment guidelines for ART eligibility over the same period. After national roll-out in 2004, local ART services were initially available from the hospital clinic [40]. Later ART services rolled out to all sixteen clinics in the health district offering ART initiation and follow-up by late 2007. Until March 2010, treatment guidelines were for ART initiation of adults with poor levels of immunity and health (CD4 cell count < 200 cells/μl or WHO stage 4) [54]. Eligibility criteria were first increased to CD4 <350 cells/μl for pregnant women and people with active tuberculosis (TB) disease, then later for all HIV-infected adults. After the end of study follow-up for this paper, eligibility criteria for ART initiation rose to CD4<500 cells/μl in January 2015 and irrespective of CD4 count from September 2016 [42]. Despite increased availability of ART, the estimated population ART coverage in HIV-positive adults in this area only increased from 31.7% in 2012 to 45% in 2017 [38,42].

Several factors may contribute to the consistently higher proportion and incidence of death among fathers compared with mothers. Mortality rates increase with age and irrespective of the effects of the HIV epidemic, fathers are on average older than mothers. Among married and regular couples belonging to the same household in the study area, men are more than 6 years older than their female partner on average [55]. There are also reasons to expect an earlier and larger impact of ART treatment on the survival of HIV-infected mothers than fathers in this population and others in South Africa. The Prevention of Mother-to-Child Transmission (PMTCT) national programme started in 2002 i.e. before the national rollout of ARV started [56]. Similar to other areas of South Africa, pregnant and recently delivered mothers have very high rates of contact with antenatal and delivery and child vaccination services [57,58]. During the study period therefore, not only were HIV-infected mothers more likely than HIV-infected fathers to have been tested for HIV, there would have been earlier improvements in the average time from HIV infection to ART eligibility and survival for women on ART treatment. A prospective study of a large cohort of HIV-infected pregnant women in the study area (2010–2014) showed that the majority of women (96%) were either on lifelong ART or ART prophylaxis within 6 months of the first antenatal visit [57]. Men’s poorer levels of engagement with healthcare generally, and specifically with HIV care, has been well documented in the study area and KwaZulu-Natal [22,42,59]. Compared with women, HIV-infected men are more likely to have a late HIV diagnosis, delay in seeking healthcare, and not remain engaged [60].

In the period before ART rollout, increasing trends in adult mortality in HIV-infected and HIV-uninfected adults and, as a result in maternal, paternal and double orphan prevalence and incidence, were reported in rural study populations in Malawi and Tanzania (1998–2004) [19] and Zimbabwe (1998–2003) [61] where comparable demographic and HIV surveillance was undertaken. The levels of all orphanhood indicators reported in the South African study population were however, much higher than those in the other rural study populations. In the years after the implementation of national HIV treatment programmes across the region, we expect more variation to have emerged in the pattern and timing of reversals in orphanhood due to differences in the underlying HIV prevalence between populations, the timing of ART rollout and variations in the extent of ART coverage [62,63]. Few comparable data in other African communities are available on changes in orphan incidence and prevalence trends before and after ART rollout. A similar pattern of decline in orphanhood in the Rakai study population in Uganda following HIV treatment was reported [64]. The prevalence and incidence of children (0–14 years) with one or both parents deceased declined significantly pre-HIV care period (2001–2003) levels of 17.2% and 2.10 ⁄ 100 person years respectively to 12.6% and 1.07 ⁄ 100 person years after HIV care was expanded (2006–2009).

The demographic surveillance data analysed in this study are of high quality and provide very detailed information on all children and adults belonging to households in this rural community over a 15-year period. There are two particularly useful features of ACDIS data for orphanhood studies: i) the deliberate distinction of biological mothers and fathers from non-biological parents and from non-parental primary carers; and, ii) the follow-up of biological parents who are not resident with their child in the study area, for example, as labour migrants [65,66]. Thus, our estimates of orphanhood are reliable and robust, as well as generalizable to other communities in KwaZulu-Natal with similar demographic and health characteristics. Some limitations remain. Although efforts were made to maximize the completeness and ensure the quality of repeated rounds of parental survival data [65,66], ‘missing’ or erroneous information about parental survival may result in under- or overestimations of orphanhood in this population. An ‘adoption effect’ in which interviewers or respondents report information about non-biological rather than biological parents has been described elsewhere [65]. In South Africa, Udjo (2011) has also suggested that African households are more likely to over-report paternal deaths than other population groups [67]. In this study however, the effects of response bias on orphanhood estimates are likely to be small given the numerous visits to households to update ACDIS information and, specific attention in questionnaire design and interviewer training. Orphanhood estimates could also be biased by parents, particularly fathers, whose survival status was not known for the whole period of follow-up [68]. However, although we expect rates of mortality to be higher in parents of unknown survival than in parents with complete data, parental survival status was unknown for only 0–2% of children at each mid-year time point. We therefore, grouped children with unknown parental survival status together with children known to be orphaned. This approach is similar to that used by UNICEF and other agencies in defining children with one deceased parent as an orphan irrespective of the survival status of the other parent [69]. This study has implications for future research on children and parents in communities with high HIV prevalence. Our results, together with the findings of other recent longitudinal studies of HIV and adult mortality [22,23] suggest that we can anticipate further gains in life-expectancy of HIV-infected adults and a continued decline in HIV/AIDS as a cause of premature adult death. Given the scale of the HIV epidemic in rural KwaZulu-Natal, there is also likely to be an increase in the number of children living with HIV-infected parents and carers for some years to come. However, the multiple determinants of overall and age-specific fertility and mortality means less certainty in the future levels of different orphanhood types [7].

The value of continuing to collect and measure orphanhood in longitudinal African population cohorts is also given emphasis by recent studies highlighting important discrepancies between the international modelled orphanhood prevalence estimates reported by the United National Programme on HIV/AIDS (UNAIDS) and similar estimates derived from survey and census data [70]. There is no current consensus about how changes in the HIV epidemic such as age and sex patterns of HIV incidence and changes in HIV treatment coverage are affecting differences in orphanhood estimates or which statistical methods are appropriate for adjusting the modelled estimates [71].

The findings of this population-based study have implications for South Africa’s formal statutory policies and social assistance programmes targeted towards orphans such as the Foster Care Grant (FCG). FCGs are a non-means tested cash payment to foster carers with legal custody of a child <18 years. Widely perceived as a *de facto* orphan grant [72], in reality only a small proportion of orphans are subject to foster care court orders [73]. In 2014, 500,000 FCGs were paid to foster carers (80% of whom were caring for orphaned children). The FCG was provided to 7.1% and 1.2% of carers for maternal and paternal orphans compared with one in three carers of double orphans (33.8%) [74]. The most commonly accessed form of assistance is the child support grant (CSG), a means-tested, unconditional grant aimed at reducing poverty and promoting investment in poor children [48]. However, parental and non-parental carers of children who are single orphans do not currently have preferential access to the CSG [75]. Government efforts to improve the capacity and delivery of South Africa’s social welfare system including the foster care arrangements and financial assistance for orphans, are ongoing [76]. Analyses of detailed, longitudinal population-based data can make a valuable contribution in ensuring statutory definitions and eligibility criteria align more closely with the diversity of residential, parenting and care arrangements in which children live [65,75,77].

In the last decade, policy and research focusing specifically on orphans and childhood adversity in South Africa have increasingly concentrated on children affected by HIV and AIDS (See for example, [78]). Related intervention programmes typically include targeted interventions to prevent sexual violence and HIV acquisition in orphans affected by HIV and AIDS, especially during adolescence [3,78]. Although the incidence of HIV/AIDS and TB maternal and paternal deaths was still higher than any other causes among older children and adolescents by 2014, it is problematic to overlook children experiencing parental deaths from other causes, particularly with respect to the impacts on early development in younger children. For example, the challenging situations faced by children and households who experience a sudden and/or violent adult death of a family member, may require specialist support. Studies conducted before HIV treatment was available in this study population described significant differences in the impact of parental death on health and wellbeing outcomes of orphans depending on the cause of parental death and the child and family circumstances before and after the death [9,15]. Consequently there is a need to continuously re-evaluate the value and effectiveness of support, particularly in relation to education, training and entry into employment, for children, adolescents and possibly young adults, who are affected by parental illness and death [3,12,79].

In conclusion, these empirical findings demonstrate that widespread availability of HIV treatment has made a profound contribution to checking and reversing mortality in mothers and fathers of young children and adolescents. Therefore, the bleakest forecasts of orphanhood levels based on orphan incidence in the 1990s and early 2000s have not been realised. However, given the dynamic and complex epidemiological and demographic changes in South Africa and other countries with HIV epidemics, we should continue to monitor the nature and level of impacts of all causes of parental ill-health and mortality in order to ensure that programme and policy responses best meet the needs of all children and adolescents.

## Supporting information

S1 Table Orphanhood prevalence by orphan status and age group in children and adolescents <20 years, ACDIS, 2000, 2005, 2010, 2014. (DOCX)

S2 Table Maternal and paternal orphaning incidence (per 1,000 PYO) by orphan status and age group in children and adolescents <20 years, 2000, 2005, 2010, 2014. (DOCX)
