# Supplementary material for: Trends in the prevalence and incidence of orphanhood in children and adolescents <20 years in rural KwaZulu-Natal South Africa, 2000-2014
Source: PLoS One. 2020 Nov 24;15(11):e0238563. doi: 10.1371/journal.pone.0238563 (PMC7685426; doi:10.1371/journal.pone.0238563)
Supplement: S1 Table — (DOCX) [file pone.0238563.s001.docx]

S1 Table. Orphanhood prevalence by orphan status and age group in children and adolescents <20 years, ACDIS, 2000, 2005, 2010, 2014.

| **Indicator of orphan status** | | | | | | | | | | |
| --- | --- | --- | --- | --- | --- | --- | --- | --- | --- | --- |
| **Year** | **Age** | **Maternal** | | **Paternal** | | **Double** | | **One or both parents died** | | **Overall**^1^ |
|  |  | %  (95% CI) | n | %  (95% CI) | n | %  (95% CI) | n | %  (95% CI) | n | N |
|  |  |  |  |  |  |  |  |  |  |  |
| **2000** | ***0-4*** | 2.2  (2.0-2.6) | 191 | 14.7  (14.0-15.5) | 1,265 | 1.2  (1.0-1.5) | 105 | 18.2  (17.4-19.1) | 1,561 | 8,584 |
|  | ***5-9*** | 4.0  (3.6-4.4) | 366 | 15.6  (14.9-16.4) | 1,427 | 2.8  (2.5-3.2) | 258 | 22.4  (21.6-23.3) | 2,051 | 9,162 |
|  | ***10-14*** | 4.9  (4.5-5.4) | 447 | 19.6  (18.8-20.4) | 1,793 | 4.7  (4.3-5.2) | 426 | 29.2  (28.3-30.1) | 2,666 | 9,156 |
|  | ***15-19*** | 5.4  (4.9-5.9) | 413 | 23.4  (22.5-24.4) | 1,827 | 7.5  (6.9-8.1) | 579 | 36.3  (35.2-37.3) | 2,819 | 7,775 |
|  | ***0-19*** | 4.1  (3.9-4.3) | 1,417 | 18.2  (17.8-18.6) | 6,312 | 4.0  (3.8-4.2) | 1,368 | 26.3  (25.8-26.7) | 9,097 | 34,677 |
|  |  |  |  |  |  |  |  |  |  |  |
| **2005** | ***0-4*** | 3.5  (3.1-3.9) | 273 | 18.0  (17.2-18.8) | 1,455 | 1.2  (1.0-1.5) | 98 | 22.6  (21.7-23.6) | 1,826 | 8,056 |
|  | ***5-9*** | 6.8  (6.3-7.3) | 575 | 16.4  (15.6-17.2) | 1,402 | 4.0  (3.6-4.4) | 340 | 27.1  (26.2-28.1) | 2,317 | 8,493 |
|  | ***10-14*** | 9.4  (8.8-10.1) | 846 | 20.3  (19.5-21.1) | 1,807 | 7.3  (6.7-7.8) | 655 | 37.0  (36.0-38.0) | 3,308 | 8,924 |
|  | ***15-19*** | 9.1  (8.5-9.8) | 745 | 23.2  (22.3-24.1) | 1,893 | 9.6  (8.9-10.2) | 773 | 41.9  (40.8-42.9) | 3,411 | 8,148 |
|  | ***0-19*** | 7.2  (7.0-7.5) | 2,439 | 19.4  (19.0-19.9) | 6,557 | 5.5  (5.3-5.8) | 1,866 | 32.2  (31.7-32.7) | 10,862 | 33,621 |
|  |  |  |  |  |  |  |  |  |  |  |
| **2010** | ***0-4*** | 3.5  (3.2-4.0) | 314 | 16.9  (16.2-17.7) | 1,537 | 1.4  (1.2-1.7) | 127 | 21.9  (21.1-22.8) | 1,978 | 9,028 |
|  | ***5-9*** | 6.9  (6.3-7.4) | 557 | 17.5  (16.7-18.3) | 1,433 | 3.9  (3.5-4.3) | 322 | 28.2  (27.3-29.2) | 2,312 | 8,233 |
|  | ***10-14*** | 9.3  (8.7-9.9) | 766 | 22.9  (22.0-23.8) | 1,867 | 9.4  (8.8-10.1) | 775 | 41.6  (40.5-42.7) | 3,408 | 8,190 |
|  | ***15-19*** | 9.6  (9.0-10.3) | 759 | 27.2  (26.2-28.2) | 2,151 | 16.0  (15.2-16.8) | 1,258 | 52.8  (51.7-53.9) | 4,168 | 7,921 |
|  | ***0-19*** | 7.2  (7.0-7.5) | 2,396 | 21.0  (20.5-21.4) | 6,988 | 7.5  (7.2-7.7) | 2,482 | 35.6  (35.1-36.2) | 11,866 | 33,372 |
|  |  |  |  |  |  |  |  |  |  |  |
|  |  |  |  |  |  |  |  |  |  |  |
| **2014** | ***0-4*** | 2.5  (2.2-2.9) | 184 | 14.2  (13.5-15.0) | 1,053 | 1.4  (1.1-1.7) | 102 | 18.1  (17.2-19.0) | 1,339 | 7,442 |
|  | ***5-9*** | 6.1  (5.6-6.7) | 507 | 13.6  (12.8-14.3) | 1,125 | 3.1  (2.7-3.5) | 257 | 22.8  (21.9-23.7) | 1,889 | 8,304 |
|  | ***10-14*** | 8.3  (7.6-8.9) | 568 | 21.8  (20.9-22.8) | 1,515 | 8.0  (7.4-8.6) | 554 | 38.1  (36.9-39.2) | 2,637 | 6,970 |
|  | ***15-19*** | 9.4  (8.7-10.1) | 668 | 26.9  (25.9-28.0) | 1,890 | 14.3  (13.5-15.1) | 992 | 50.6  (49.5-51.8) | 3,550 | 7,017 |
|  | ***0-19*** | 6.5  (6.2-6.8) | 1,927 | 18.8  (18.4-19.3) | 5,583 | 6.4  (6.2-6.7) | 1,905 | 31.8  (31.2-32.3) | 9,415 | 29,733 |

Table S1 Footnotes:

^1.^ Children were excluded from the analytical mid-year populations of resident children <20 years if no valid observation of parental survival status was available. The numbers excluded and the percentage of the total surveillance population aged <20 years were 198 (0.6%) in 2000, 617 (1.79 %) in 2005, 668 (1.9%) in 2010 and, 476 (1.6%) in 2014.
