# Supplementary material for: Trends in the prevalence and incidence of orphanhood in children and adolescents <20 years in rural KwaZulu-Natal South Africa, 2000-2014
Source: PLoS One. 2020 Nov 24;15(11):e0238563. doi: 10.1371/journal.pone.0238563 (PMC7685426; doi:10.1371/journal.pone.0238563)
Supplement: S2 Table — (DOCX) [file pone.0238563.s002.docx]

S2 Table. Maternal and paternal orphaning incidence (per 1,000 PYO) by orphan status and age group in children and adolescents <20 years, 2000, 2005, 2010, 2014

|  |  |  | **Maternal orphaning incidence**^1^ | | |  | **Paternal orphaning incidence** | | |
| --- | --- | --- | --- | --- | --- | --- | --- | --- | --- |
| **Year** | **Age** | **PYO (1000s)** | **Maternal deaths** | **Maternal incidence**  **(95% CI)**^3^ | **% Unknown maternal status**^2^ | **PYO (1000s)** | **Paternal deaths** | **Paternal incidence**  **(95% CI)** | **% Unknown paternal status**^4^ |
| 2000 | 0-4 | 9.8 | 80 | 8.1 (6.5-10.1) | 1.7 | 5.8 | 105 | 18.1 (14.9-21.9) | 1.0 |
|  | 5-9 | 9.5 | 142 | 15.0 (12.7-17.7) | 1.4 | 6.2 | 140 | 22.6 (19.2-26.7) | 0.8 |
|  | 10-14 | 9.1 | 143 | 15.7 (13.3-18.5) | 1.2 | 6.0 | 162 | 26.9 (23.0-31.3) | 0.6 |
|  | 15-19 | 8.1 | 129 | 15.8 (13.3-18.8) | 0.9 | 5.3 | 183 | 34.6 (29.9-40.0) | 0.5 |
|  | 0-19 | 36.6 | 494 | 13.5 (12.4-14.8) | 1.4 | 23.3 | 590 | 25.3 (23.3-27.4) | 0.7 |
| 2005 | 0-4 | 8.9 | 84 | 9.5 (7.6-11.7) | 1.3 | 4.0 | 64 | 15.9 (12.4-20.3) | 1.2 |
|  | 5-9 | 7.9 | 137 | 17.2 (14.6-20.4) | 1.6 | 4.6 | 122 | 26.3 (22.0-31.4) | 1.3 |
|  | 10-14 | 7.6 | 151 | 19.9 (16.9-23.3) | 1.3 | 4.8 | 142 | 29.7 (25.2-35.0) | 1.0 |
|  | 15-19 | 7.2 | 156 | 21.8 (18.6-25.5) | 1.2 | 4.6 | 152 | 33.1 (28.3-38.9) | 1.1 |
|  | 0-19 | 31.6 | 528 | 16.7 (15.3-18.2) | 1.3 | 18.0 | 480 | 26.6 (24.4-29.1) | 1.1 |
| 2010 | 0-4 | 9.5 | 60 | 6.3 (4.9-8.2) | 1.3 | 10 | 52 | 15.0 (11.4-19.7) | 1.2 |
|  | 5-9 | 7.7 | 91 | 11.9 (9.7-14.6) | 1.6 | 3.5 | 65 | 18.7 (14.7-23.8) | 1.6 |
|  | 10-14 | 6.5 | 81 | 12.5 (10.1-15.6) | 1.2 | 3.6 | 90 | 24.8 (20.2-30.5) | 1.4 |
|  | 15-19 | 6.2 | 78 | 12.5 (10.0-15.7) | 1.2 | 3.7 | 99 | 26.6 (21.9-32.4) | 0.8 |
|  | 0-19 | 29.8 | 310 | 10.4 (9.3-11.6) | 1.3 | 14.3 | 306 | 21.4 (19.1-24.0) | 1.2 |
| 2014 | 0-4 | 8.1 | 33 | 4.1 (2.9-5.7) | 1.1 | 2.1 | 19 | 9.2 (5.9-14.4) | 1.0 |
|  | 5-9 | 8.1 | 62 | 7.7 (6.0-9.8) | 1.5 | 3.2 | 34 | 10.6 (7.6-14.8) | 1.1 |
|  | 10-14 | 5.9 | 51 | 8.7 (6.6-11.4) | 1.0 | 2.7 | 32 | 11.7 (8.2-16.5) | 0.8 |
|  | 15-19 | 5.6 | 41 | 7.3 (5.4-9.9) | 1.1 | 3.1 | 51 | 16.5 (12.5-21.7) | 0.8 |
|  | 0-19 | 27.7 | 187 | 6.8 (5.9-7.8) | 1.2 | 11.1 | 136 | 12.2 (10.3-14.5) | 0.9 |

Table 2 Footnotes:

^1.^ Maternal (or paternal) orphaning incidence is estimated for the year period for resident children <20 years whose mothers (or fathers) were alive at the start of the year; and is expressed 1000 person (i.e. child) years of observation.

^2.^ In the table the percentage of all resident children and adolescents <20 years is shown for each year.

^3.^ Children and adolescents <20 were excluded from the calculation of annual estimate of orphaning if the survival of the mother was not known at the start of the year. The numbers of children not included were 502 children and adolescents (1.36%) in 2000, 427 (1.33 %) in 2005, 406 (1.35%) in 2010 and 336 (1.21%) in 2014.

^4.^ The number and percentage of all children and adolescents <20 years excluded in the annual calculation of paternal orphaning incidence were 172 (0.73%) in 2000, 210 (1.14%) in 2005, 178 (1.23%) in 2010 and 103 (0.92%) in 2014.

Figure 2. Maternal and paternal orphaning incidence (per 1,000 PYO) by orphan status and age group in children and adolescents <20 years linked to their parental records, ACDIS, 2000, 2005, 2010, 2014

2a. Maternal orphaning incidence

2b. PaternJual orphaning incidence
